# Requirments for primary human hepatocyte

**DOI:** 10.1111/cpr.13147

**Published:** 2021-12-22

**Authors:** Zhaoliang Peng, Jiaying Wu, Shijun Hu, Aijin Ma, Lei Wang, Nan Cao, Yu Zhang, Qiyuan Li, Junying Yu, Shufang Meng, Tao Na, Xiaolei Shi, Mei Li, Huadong Liu, Linguang Qian, E Tian, Feng Lin, Jiani Cao, Yaojin Peng, Huanxin Zhu, Lingmin Liang, Jie Hao, Tongbiao Zhao, Xin Cheng, Guoyu Pan

**Affiliations:** ^1^ Shanghai Institute of Materia Medica Chinese Academy of Sciences Shanghai China; ^2^ Center for Excellence in Molecular Cell Science Chinese Academy of Sciences Shanghai China; ^3^ Beijing Institute for Stem Cell and Regenerative Medicine Beijing China; ^4^ Beijing Technology and Business University Beijing China; ^5^ National Stem Cell Resource Center State Key Laboratory of Stem Cell and Reproductive Biology Institute of Zoology Institute for Stem Cell and Regeneration Chinese Academy of Sciences Beijing China; ^6^ University of Chinese Academy of Sciences Beijing China; ^7^ Zhongsan School of Medicine Sun YAT‐SEN University Guangzhou China; ^8^ Shenzhen Huada Gene Research Institute Beijing China; ^9^ Nuwacell Co., Ltd Anhui China; ^10^ National Institutes for Food and Drug Control Beijing China; ^11^ Nanjing Drum Tower Hospital Nanjing China; ^12^ Corning Life Sciences (Wujiang) Co., Ltd Shanghai China; ^13^ Shanghai Transheep Biotechnology Co., Ltd Shanghai China; ^14^ Shanghai Hexaell Biotechnology Co., Ltd Shanghai China

**Keywords:** primary human hepatocyte, quality control, standard

## Abstract

‘Requirements for Primary Human Hepatocyte’ is the first set of guidelines on Primary Human Hepatocyte in China, jointly drafted and agreed upon by experts from the Chinese Society for Stem Cell Research. This standard specifies the technical requirements, test methods, test regulations, instructions for use, labelling requirements, packaging requirements, storage requirements and transportation requirements for Primary Human Hepatocyte, which is applicable to the quality control for Primary Human Hepatocyte. It was originally released by the China Society for Cell Biology on 9 January 2021. We hope that publication of these guidelines will promote institutional establishment, acceptance and execution of proper protocols and accelerate the international standardization of Primary Human Hepatocyte for applications.

## 1 SCOPE

This document specifies the technical requirements, test methods and regulations, instructions for use, labelling requirements, packaging requirements, storage requirements, transportation requirements and waste disposal requirements for primary human hepatocytes.^1^

This standard is applicable for the production and testing of primary human hepatocytes after birth.

## 2 NORMATIVE REFERENCES

The following content constitutes indispensable articles of the standard through normative reference. For dated references, only the edition cited applies. For undated references, only the latest edition (including all amendments) applies.

WS213 *Diagnosis for hepatitis C*.

WS213 *Diagnosis for syphilis*.

WS213 *Diagnosis for HIV*/*AIDS*.

*Pharmacopoeia* *of the People's Republic of China*.

*National* *Guide to Clinical Laboratory Procedures*.

## 3 TERMS AND DEFINITIONS

The following terms and definitions apply to this document.

### 3.1 Primary human hepatocyte

The hepatic parenchymal cells are isolated from the human liver, with the typical morphological, molecular and functional characteristics (including, but not limited to, synthesis, secretion, metabolism and detoxification) of hepatocytes.

### 3.2 Hepatocyte

A polygonal epithelial parenchymal cell of the liver, which has the centred, single or dual or multiple nuclei that are enriched for euchromatins. The hepatocytes are aligned as cords in the liver, with their basolateral surfaces facing the sinusoidal endothelial cells, and the apical surfaces between neighbouring cells forming canaliculi through tight junctions to collect and transport bile acids and bile salts. The hepatocytes feature major physiological functions, including but not limited to synthesis, secretion (both exocrine and endocrine), metabolism, detoxification and storage.

## 4 ABBREVIATIONS

The following abbreviations are applicable for this document.

ALB: ALBUMIN.

BEI: Biliary Excretion Index.

CYP: Cytochrome P450.

EBV: Epstein‐Barr Virus.

HAV: Hepatitis A Virus.

HBV: Hepatitis B Virus.

HCMV: Human Cytomegalovirus.

HCV: Hepatitis C Virus.

HIV: Human Immunodeficiency Virus.

HNF4A: Hepatocyte Nuclear Factor 4 Alpha.

HTLV: Human T‐lymphotropic Virus.

TP:Treponema Pallidum.

## 5 TECHNICAL REQUIREMENTS

### 5.1 Source materials and ancillary materials

The collection of human biological source material (source material) shall comply with domestically recognized ethics and local laws and regulations.

Written and valid informed consent shall be signed by the donor. The content of informed consent shall include but not be limited to the research purpose, potential research and clinical diagnosis application under appropriate conditions feedback on unexpected discoveries potential commercial value and other issues affiliated to the research. Mechanisms for protecting the personal data and privacy of donors shall be established.

The cell research and production organizations shall establish and implement cell donor evaluation standards, and establish collection methods, transportation standards, handover standards and storage standards to ensure the safety of donors and cells. The source material and the derivative cells shall be accompanied with detailed documentation on acquisition methods, source and relevant clinical information, including but not limited to the donor's general information, past medical history and family history. The past medical history and family history shall be collected in detail for the information related to genetic diseases. The information of the donor's ABO blood type and the human leukocyte antigen (HLA) type I and type II classification shall be collected.

The origin of cells shall be traceable by referring to the relevant informed consent and/or their genome and functional data.

Ancillary materials such as culture medium and growth factors shall meet the corresponding quality control requirements. The ancillary materials shall be inspected and laboratory tested if necessary.

When using animal serum, they shall be demonstrated to be of low risk of viral contamination and donor testing performed where appropriate. Serum from animals in geographical regions with known prion epidemics (e.g. bovine spongiform encephalopathy) shall be prohibited.

If human blood components are used in the culture medium, including but not limited to albumin, transferrin and various cytokines, the source, batch number and quality verification reports shall be provided. State‐approved meterials shall be used where feasible.

The source material donors should be screened for HIV, HAV, HBV, HCV, HTLV and TP, and the results should be recorded.

The isolation and acquisition of cells shall be performed in a primary (or secondary) biosafety laboratory.

### 5.2 Primary quality attributes

#### 5.2.1 Cell morphology

Primary human hepatocyte cultured in 2D conditions shall exhibit a polygonal morphology with a diameter of 20–30 µm under the optical microscope. Cells can have single, dual or multiple nuclei that are large and round in shape. Tight junctions and Bile duct can be found between cells in the adherent cultures.

#### 5.2.2 Cell viability

The cell viability shall be ≥70% after resuscitation.

#### 5.2.3 Cell markers

The expression of ALB and HNF4A shall be ≥90% of the cell population.

#### 5.2.4 Albumin secretion

The Albumin secretion shall be ≥800 ng/(10^6^ cells × 24 h).

#### 5.2.5 Drug metabolism function

The representative drug metabolism ability of PHHs (e.g. CYP3A4, testosterone as the substrate) indicated by the intrinsic clearance rate shall be ≥100 μl/(h × 10^6^ cells).

#### 5.2.6 Bile secretion (applicable to adherent cells only)

The BEI index (d8‐TCA as the substrate) shall be ≥30%.

#### 5.2.7 Microorganisms

Primary human hepatocytes shall be negative for bacteria, fungi, mycoplasma, HIV, HAV, HBV, HCV, HTLV, EBV, HCMV and TP.

### 5.3 Process control

#### 5.3.1 Cell isolation

During the cell isolation process, cross‐contamination and confusion between donors, tissues and cells shall be avoided and a risk management strategy shall be developed.

During the process of culture and expansion after cell isolation, the passage and the cells name shall be clearly specified and the operation date, culture conditions, operators' names or initials shall be indicated.

#### 5.3.2 Cell isolation process and cell identification

The equipment, the culture system and the operation steps used for cell isolation shall be clarified. The standard operating procedure for cell isolation shall be established for reproducibility.

The isolated cells shall have the defined morphological, molecular and functional characteristics listed in 5.2.

#### 5.3.3 Cryopreservation

Cryopreserved PHHs shall be clearly labelled with the cell name, the culture conditions, the passage number, the operators' names or initials and the cryopreservation date. Cryopreserved cells shall have the same unique identification used during the processes of collection, isolation and culturing.

The cryopreservation procedure shall follow the known principles of cryopreservation of mammalian cells and shall be recorded according to the relevant regulations.

#### 5.3.4 Resuscitation

The resuscitation process shall be as short as possible to ensure the optimal viability and maintenance of biological functions of PHHs.

The cell information, including but not limited to the cell name, the batch name, the passage number, the culture conditions, operators' names or initials and resuscitation date, shall be documented and recorded.

#### 5.3.5 Identification of cell STR

The short tandem repeat (STR) signature of PHHs shall be consistent with that of the donor.

## 6 TEST METHODS

### 6.1 Cell morphology

Observe the morphology of cells grown under 2D conditions in vitro using a microscope.

### 6.2 Cell viability

The method in Appendix S1 shall be followed.

### 6.3 Cell markers

The method in Appendix S2 shall be followed.

### 6.4 Albumin secretion

The method in Appendix S3 shall be followed.

### 6.5 Function of drug metabolism enzyme

The method in Appendix S4 shall be followed.

### 6.6 Bile secretion index

The method in Appendix S5 shall be followed.

### 6.7 Microorganisms

The microorganism reports shall be provided by the manufacturer.

#### 6.7.1 Fungi

The method "1101 Sterility Test" in Pharmacopoeia of the *People's Republic of China* shall be followed.

#### 6.7.2 Bacteria

The method "1101 Sterility Test" in "Pharmacopoeia of the *People's Republic of China*" shall be followed.

#### 6.7.3 Mycoplasma

The method "3301 Sterility Test" in Pharmacopoeia of the *People's Republic of China* shall be followed.

#### 6.7.4 HIV

The method in WS 293 shall be followed.

#### 6.7.5 HAV

The method in *National Guide to Clinical Laboratory Procedures* shall be followed.

#### 6.7.6 HBV

The method in National Guide to Clinical Laboratory Procedures shall be followed.

#### 6.7.7 HCV

The method in WS 213 shall be followed.

#### 6.7.8 HTLV

The method in National Guide to Clinical Laboratory Procedures shall be followed.

#### 6.7.9 EBV

The method in National Guide to Clinical Laboratory Procedures shall be followed.

#### 6.7.10 HCMV

The method in *National Guide to Clinical Laboratory Procedures* shall be followed.

#### 6.7.11 TP

The method in WS 273 shall be followed.

## 7 INSPECTION RULES

### 7.1 Sampling method

Cells produced from the same production line, the same source, the same passage and the same method are considered to be the same batch.

Three smallest packaging units shall be randomly sampled from the same batch.

### 7.2 Quality inspection and release

Each batch of products shall be subject to quality inspection before release, and an inspection report shall be attached.

The quality inspection items shall include all the attributes specified in 5.2.

### 7.3 Review inspection

The review inspection shall be performed by professional cytological testing institutions/laboratories as necessary.

### 7.4 Decision rules

The PHH preparations that meet all requirements in 5.2 for the quality inspection for release are considered to be qualified. The PHH preparations that fail to meet one or more requirements in 5.2 or the quality inspection for release are considered to be unqualified.

The PHH preparations that meet all requirements in 5.2 for the quality review inspection are considered to be qualified. The PHH preparations that fail to meet one or more requirements in 5.2 for the review inspection are considered to be unqualified.

#### 7.4.1 INSTRUCTIONs FOR USAGE

The instructions for usage shall include, but are not limited to:

- Product name;
- Cell source;
- Cell number;
- Production date;
- Lot number;
- Production organization;
- Storage conditions;
- Shipping conditions;
- Operation manual;
- Execution standard number;
- Manufacturing address;
- Contact information;
- Postal code;
- Matters that deserve attention.

Note: The information on endotoxin concentration in the products can be provided by the manufacturer upon request.

## 8 LABELS

The label shall include but not limited to:

- Product name;
- Cell source;
- Cell number;
- Lot number;
- Production organization;
- Production date.

## 9 PACKAGE, STORAGE AND TRANSPORTATION

### 9.1 Package

The appropriate materials and containers shall be selected to ensure the maintenance of the primary quality attributes of PHHs.

### 9.2 Storage

The principles and standard operating procedures for the storage and management of PHHs should be established and implemented. Detailed information, including but not limited to all documentation of PHHs, the application requests to cell storage organization and ethical reviews shall be recorded.

The application requests for primary human hepatocytes should be submitted to and approved by the cell storage organization.

The primary human hepatocytes stored in the cell bank shall be in accordance with the management requirements for cell banks.

Stored at temperatures below −130°C.

### 9.3 Transportation

The appropriate mode of transportation and transportation conditions shall be selected to ensure the preservation of the optimal biological characteristics, safety, stability and efficacy of primary human hepatocytes.

The transportation of primary human hepatocytes shall consider the following factors, including but not be limited to the cell characteristics, the container, the transportation routes, the transportation conditions, the transportation equipment, the transportation methods, the transportation risks and risk mitigation measures.

The management of transportation conditions shall include, but not be limited to, temperature range, vibration, contamination, equipment performance and packaging.

The transportation documentation shall include, but is not limited to, information on the model and condition of transportation, the transportation routes, the duration time, the personnel, the shipping address and the information of primary human hepatocytes.

Cryopreserved cells should be transported in dry ice or at temperatures below −130°C, and non‐cryopreserved cells should be transported at 2−8°C.

## 10 WASTE DISPOSAL

Management documents for disposal of PHHs shall be established, and the management specifications shall be strictly implemented and recorded in detail.

Disqualified/discarded PHHs or their raw source materials that arise during research and production shall be disposed of according to legal and/or ethical regulations.

## CONFLICT OF INTEREST

No potential conflicts of interest are disclosed.

## AUTHOR CONTRIBUTIONS

GP and CX contributed to conception and design. ZP and JW drafted and revised the manuscript. SH, AM, LW, NC, YZ, QL, TY, SM, TN, XS, ML, HL, LQ, ET, FL, JC, YP, HZ, LL, JH and TZ critically read and revised the manuscript.

## Supporting information

Supplementary Material

## Data Availability

The raw data supporting the conclusions of this article will be made available by the authors.
