# Supplementary material for: Requirments for primary human hepatocyte
Source: Cell Prolif. 2021 Dec 22;55(4):e13147. doi: 10.1111/cpr.13147 (PMC9055892; doi:10.1111/cpr.13147)
Supplement: Supplementary file 1 — Supplementary Material [file CPR-55-e13147-s001.docx]

**Appendix S1**

**(Normative appendix)**

**CELL VIABILITY TEST (CELL ENUMERATION METHOD)**

**A.1 Instruments**

A.1.1 Microscope.

A.1.2 Haemocytometer.

**A.2 Reagents**

Unless otherwise stated, all reagents used shall be of analytical grade. The water used for testing shall be deionized water.

A.2.1 Phosphate buffered saline (PBS): pH 7.4.

A.2.2 The Trypan Blue solution shall be diluted to a final concentration of 0.4 % (w/v) with phosphate buffered saline.

**A.3 Procedures**

A.3.1 Preparation of cell suspension

Harvest and suspend the cells with appropriate volume of PBS (A.2.1). The density of cells in the haemocytometer shall be 20-50 cells/mm^2^.

A.3.2 Trypan Blue staining

Evenly mix the Trypan blue solution (A.2.2) with the cell suspension at a volume ratio of 1:1.

A.3.3 Cell counting

The cover glass is covered on the counting tank of haemocytometer (A.1.2). Load the haemocytometer with 10 μL of the trypan blue-labelled sample. Make sure the entire chamber is filled with the testing sample. Stand for 30 seconds, and count the stained cells and the total number of cells respectively.

For the 16 × 25 counting chamber, use the four 1 mm^2^ medium squares at the top left, top right, bottom left, and bottom right of the chamber (i.e., 100 small squares) for counting.

For the 25 × 16 counting chamber, use the five 1 mm^2^ medium squares at the top left, top right, bottom left, bottom right, and center of the chamber (i.e., 80 small squares) for counting.

When there are cells on the lines of the large square, only cells on the top line and left line of the large square can be counted (or alternatively only cells on the bottom line and right line).

Repeat once following the aforementioned procedure.

**A.4 Calculation and Analysis**

Cell viability is calculated according to equation (A.1):

X = (M-S) / M ×100% (A.1)

In the equation:

X— viability of cells

M—total number of cells

S—number of stained cells

The viability of cells is the mean of two or three duplicate samples. Two or three independent cell viability tests shall be performed on the same sample. The mean value of two or three independent viability tests is recorded as the viability of cells.

**A.5 Accuracy**

The absolute difference value between the two independent tests, under the same conditions, shall not exceed 20% of their arithmetic mean.

**Appendix S2**

**(Normative appendix)**

**DETECTION OF CELL MARKERS (FLOW CYTOMETRY)**

**B.1 Instruments**

B.1.1 Flow cytometer.

B.1.2 Bench-top centrifuge.

**B.2 Reagents**

Unless otherwise stated, all the reagents used shall be of analytical grade. The water used in the experiment shall be Grade 1 water as stipulated in GB/T 6682.

B.2.1 Phosphate buffered saline (PBS): pH 7.4.

B.2.2 Paraformaldehyde (PFA).

B.2.3 Bovine serum albumin (BSA): Purity ≥98%.

B.2.4 Antibodies (Anti-human ALB antibody, anti-human HNF4A antibody and isotype control antibody).

B.2.5 Prepare the following solutions according to the requirements for flow cytometry: wash solution, fixing solution, permeabilization solution, and antibody dilution solution.

**B.3. Sample storage**

The wash solution and fixed samples shall be stored at 2-8°C. Antibodies shall be stored according to the manufacturer's instructions.

**B.4 Procedures**

B.4.1 Sample preparation

Collect samples by centrifuging single cell suspensions at 250 g for 3 minutes. Discard the supernatant. 1~3 × 10^5^ single cell are needed for each sample.

B.4.2 Fixation

Resuspend the cells in an appropriate volume of fixing solution and incubate for appropriate time. Wash the cell samples with an appropriate volume of wash solution for 3-5 times. Collect samples by centrifuging single cell suspensions at 250 g for 3 minutes. Discard the supernatant. Resuspend the cell samples, and store in 4°C or continue to test immediately.

B.4.3 Permeabilization

Resuspend the fixed sample with the wash solution and aliquot the cells into two independent samples, which will be used as the testing sample and the isotype control sample respectively. Centrifuge the cells, discard the supernatant and resuspend with an appropriate volume of permeabilization solution. Wash the cell samples with an appropriate volume of wash solution for 3-5 times. Collect samples by centrifuging single cell suspensions at 250 g for 3 minutes. Discard the supernatant.

B.4.4 Primary antibody incubation

Incubate the samples with the diluted anti-human ALB antibody or anti-human HNF4A antibody or corresponding isotype controls according to the manufacturer's instructions. After incubation, wash the cell samples with an appropriate volume of wash solution for 3-5 times. Collect samples by centrifuging single cell suspensions at 250 g for 3 minutes. Discard the supernatant.

B.4.5 Secondary antibody incubation

Choose appropriate secondary antibody and incubate the samples with the diluted secondary antibodies according to the manufacturer's instructions. The cell samples should be incubated with antibodies for 30 minutes at room temperature in darkness.

B.4.6 Filtering and loading

After incubation, wash the cell samples with an appropriate volume of wash solution for 3-5 times. Collect samples by centrifuging single cell suspensions at 250 g for 3 minutes. Discard the supernatant. Resuspend the samples with wash solution and then transfer the cell suspension into a flow cytometry tube by filtering the samples through a Nylon mesh with 70 μm pores. Load the samples into the flow cytometer and perform testing according to the manufacturer's instruction.

B.5 Data processing

The original data shall be processed by flow cytometry analysis software. The live cell population is defined according to the parameters of cell granularity (SSC) and light transmittance (FSC), with cell debris and other irrelevant particles being excluded. The gating of positively stained cells shall be determined by the fluorescence intensity using the isotype control as a reference. Both the negative and the positive experimental controls shall be set up for gating and following analysis.

**Appendix S3**

**(Normative appendix)**

**DETECTION of SECRETED ALBUMIN (ELISA)**

**C.1 Instruments**

C.1.1 Microplate reader.

**C.2 Reagents**

Prepare the following solutions according to the requirements for ELISA: coating solution, wash solution, blocking solution, antibody dilution solution and stop solution.

**C.3. Sample storage**

Collect the media from the primary human hepatocytes cultured for 24 h under the 2D condition. Centrifuge and collect the medium supernatant, and record the volume. Meanwhile, harvest primary human hepatocytes and count the cell number. The culture supernatant shall be stored at - 80 °C.

**C.4 Procedures**

C.4.1 Preparation of sample and standard

Dilute the sample and standards according to the manufacturer's instructions. The sample’s concentration after dilution should be within the standard range.

C.4.2 Primary antibody coating

Dilute the primary antibody according to the manufacturer’s manual. Add diluted antibody into a 96-well-plate and incubate at room temperature. Wash with wash solution for 3-5 times, and aspirate to remove the liquid.

C.4.3 Blocking

Add blocking solution into a 96-well-plate and incubate at room temperature. Wash with wash solution for 3-5 times, and aspirate to remove the liquid.

C.4.4 Incubation of sample and standards

Add the samples and the serially diluted standards into a 96-well-plate and incubate at room temperature as instructed. Wash with wash solution for 3-5 times and aspirate to remove the liquid.

C.4.5 Incubation of secondary antibody

Dilute the secondary HRP antibody according to the kit manufacturer’s manual. Add diluted antibody into a 96-well-plate and incubate at room temperature. Wash with wash solution for 3-5 times and aspirate to remove the liquid.

C.4.6 Incubation of substrate

Add TMB substrate solution. Incubate in dark at room temperature.

C.4.7 Reaction termination

Stop the reaction by adding stop solution. Measure the absorbance on microplate reader immediately.

C.5 Data Processing

The standard curve shall be prepared according to the standard concentrations and the corresponding absorbance values. The sample albumin concentration A is determined according to the standard curve. The content of the secreted ALBUMIN in the media harvested from the 24 h adherent culture of PHHs shall be calculated according to the equation (C.1):

B = A × V (C.1)

In the equation:

B—Albumin content

A—Concentration of ALBUMIN

V— Volume of medium supernatant

Mean ALBUMIN secretion of PHHs within 24 hours shall be determined according to the equation (C.2):

D = B / E (C.2)

In the equation:

D—ALBUMIN secretion. Unit: ng per 10^6^ cells per 24 hours. [ng/(10^6^ cells 🞄 24 h)]

B—ALBUMIN content.

E—Cell number.

**Appendix S4**

**(Normative appendix)**

**CELL METABOLIC FUNCTION TEST (DRUG-METABOLIZING FUNCTION ASSAY)**

**D.1 Substrate classification and mass spectrometric qualifier ions**

The substrate classification is shown in Table D1, and the mass spectrometric qualitative ion pair is shown in Table D2.

**D.2 Experiment grouping**

The experiment grouping is shown in Table D3

**D.3 Test procedure**

D.3.1 After resuscitation, seed primary human hepatocytes at a density of no less than 1×10^6^ cells/well in a 12-well or 24-well plate, add 0.5 mL of culture medium to each well, and conduct three biological replicates.

D.3.2 Add 0.5 mL of substrate dissolved in primary human hepatocyte culture medium to each well (inspection of substrate clearance, final concentration is 1 μmol/L); after mixing, take out 100 μL of cell suspension and mix it with 300 μL of ice acetonitrile, which is defined as the sample at 0 h time point.

D.3.3 Incubate the rest of the experimental samples in a constant temperature culture shaker at 37 °C at a speed of 100 r/min. At the corresponding time points, take out 100 μL of the cell suspension and mix with 300 μL of ice acetonitrile to terminate the reaction.

D.3.4 Vortex the terminated sample and centrifugate. Take out 150 μL of the supernatant for liquid chromatography tandem mass spectrometry (LC-MS/MS) assay.

**D.4 Data processing**

**D.4.1 Graphics software**

GraphPad Prism 5.0 or above or similar software is recommended.

**D.4.2 Calculation of intrinsic clearance (CL_int_）**

Take the natural logarithm of the ratio of the peak area of the sample or positive drug to the peak area of the internal standard as the ordinate, with time (h) as the abscissa, plot and perform linear regression analysis (Y=a*x+b). The intrinsic clearance rate is calculated according to formula (D.1):

CLint= (0.693/T_1/2_)×(V/M) （D.1）

In the equation:

CLint——Intrinsic clearance rate; the unit is ng per 10^6^ cells for 24 hours [ng/(10^6^ cells 🞄 24h)];
T_1/2_ (h)——0.693/K;
K——Elimination rate constant;

V——Incubation volume;

M——Number of incubated cells (in 10^6^ cells).

**Table D1. Substrate classification**

| CYP450 enzyme | Substrate | Metabolite |
| --- | --- | --- |
| CYP1A2 | Phenacetin | Paracetamol |
| CYP3A4 | Testosterone | 6β-OH testosterone |
| CYP2B6 | Bupropion | 4-OH Bupropion |

Note: The substrates and metabolites are standard substances and their purities are greater than 98%.

**Table D2. Mass spectrometric qualitative ion pair**

| Substrate | Recommended qualitative ion  pair | Metabolite | Recommended qualitative ion  pair |
| --- | --- | --- | --- |
| Phenacetin | 180/110.1 | Acetaminophen | 152.1/11.01 |
| Testosterone | 289.1/97.05 | 6β-OH Testosterone | 305/269.2 |
| Bupropion | 240.1/184 | 4-OH Bupropion | 256.1/238 |

**Table D3. Experiment grouping**

| Group | Experimental group (n=3) | Negative control (n=3) |
| --- | --- | --- |
|  | Primary human hepatocyte |  |
| Concentration of compounds | 1 μmol/L | 1 μmol/L |
| Sampling time points | Pre-0 h, Pre-4 h, 0 h, 0.5 h, 1 h, 2 h and 4 h | |
| Note 1: 4% BSA is added to all reaction systems to reduce the influence of non-specific adsorption on the experimental results.  Note 2: Pre-0 h and Pre-4 h refer to the time points when the drug is just prepared and when it is placed for 4 hours in the same environment. They both belong to the blank control group to eliminate the background of the blank control and the influence of the experimental environment.  Note 3: 0 h and 0.5 h: Sample within 0.5 h after the drug is added to the cells.  Due to the rapid elimination of testosterone, the sampling time is recommended to be 0 min, 2 min, 5 min, 10 min and 30 min (due to individual differences, the sampling time of slow-metabolizing drugs can be adjusted according to the situation). | | |

**Appendix S5**

**(Normative appendix)**

**BILE SECRETION NDEX TEST**

**E.1 Experimental principle**

After adherent culture, the primary human hepatocytes form the structure of bile canaliculi. Incubation with HBSS without Ca^2+^ can destroy the structure of bile canaliculi, thereby inducing the release of the drug in the bile canaliculi. After incubation with HBSS containing Ca^2+^, the detected accumulation in primary human hepatocytes is the sum of the drug accumulation in cells and the drug content in bile canaliculi. BEI is calculated according to formula (E.1):

BEI =(A Ca^2+^-Ca^2+^_Free_)/ Ca^2+^ ×100% （E.1）

In the equation:

ACa^2+^——Substrate concentration in primary human hepatocytes under the incubation condition of calcium-containing HBSS;

ACa^2+^_Free_——Substrate concentration in primary human hepatocytes under the incubation condition of calcium-free HBSS

The BEI assay is generally used to evaluate whether the tested drug is a substrate of an efflux transporter. Under common circumstances, when the BEI index of the test compound is greater than 10%, the compound can be considered as a substrate of the efflux transporter on bile canaliculi of primary human hepatocytes.

**E.2 Experimental purpose**

To inspect the BEI value of d8-TCA (taurocholic acid-d8 sodium salt).

**E.3 Instruments**

Triple quadrupole LC/MS, cell incubator and ultra-clean bench.

**E.4 Reagents**

Cell culture media required for cell resuscitation and culture shall be used in accordance with the corresponding requirements.

**E.5 Experimental methods**

E.5.1 Isolate primary human hepatocytes and culture them in a sandwich system, and determine the number or concentration of the cells.

E.5.2 Prepare calcium-containing and calcium-free (+/- Ca^2+^) HBSS solutions respectively.

E.5.3 Preparation of drugs

Prepare d8-TCA (5 μM) using HBSS containing Ca^2+^.

E.5.4 Discard the original medium, add HBSS with +/- Ca^2+^ and wash 3 times. Then add calcium-containing or -free HBSS culture medium, and incubate at 37 °C for 15 minutes.

E.5.5 Remove the supernatant, add the drug prepared with HBSS containing Ca^2+^ and incubate at 37 °C for 15 minutes.

E.5.6 Discard the drug solution, wash 3 times with pre-cooled PBS to stop the reaction.

E.5.7 After sealing with parafilm, store the samples at -80 °C or directly process them for mass spectrometry assay.

**E.6 Data processing method**

Calculate the drug concentrations of each group according to the standard curve, and normalize with the corresponding protein concentrations.

Sample concentration/corresponding protein concentration = sample concentration after calibration.

Take the average of the groups with or without calcium to calculate BEI.
